# Preliminary results from a Phase I/II study of epacadostat (incb024360) in combination with pembrolizumab in patients with selected advanced cancers

**DOI:** 10.1186/2051-1426-3-S2-O7

**Published:** 2015-11-04

**Authors:** Tara C Gangadhar, Omid Hamid, David C Smith, Todd M Bauer, Jeffrey S Wasser, Jason J Luke, Ani S Balmanoukian, David R Kaufman, Yufan Zhao, Janet Maleski, Lance Leopold, Thomas F Gajewski

**Affiliations:** 1 Abramson Cancer Center of the University of Pennsylvania, Philadelphia, PA, USA; 2 The Angeles Clinic and Research Institute, Los Angeles, CA, USA; 3 University of Michigan, Ann Arbor, MI, USA; 4 Sarah Cannon Research Institute, Tennessee Oncology, PLLC, Nashville, TN, USA; 5 University of Connecticut Health Center, Farmington, CT, USA; 6 University of Chicago, Chicago, IL, USA; 7 Merck & Co., Inc., Kenilworth, NJ, USA; 8 Incyte Corporation, Wilmington, DE, USA

## Background

Indoleamine 2,3-dioxygenase 1 (IDO1) is a tryptophan-catabolizing enzyme that is expressed in many cancers and induces immune tolerance by suppressing T cell responses. Epacadostat is a potent, selective oral inhibitor of IDO1. A dose-escalation study of epacadostat with ipilimumab in patients with advanced melanoma showed favorable ORR, disease control rate (DCR), and PFS in immunotherapy-naïve patients [1]. Preliminary data of epacadostat with pembrolizumab in patients with selected advanced cancers are reported.

## Methods

This is an ongoing dose-escalation and dose-expansion study of epacadostat with pembrolizumab in patients with Stage IIIB, IV, or recurrent NSCLC, melanoma, transitional cell carcinoma (TCC), RCC, endometrial adenocarcinoma (EA), or SCCHN with a 3+3+3 Phase I design (NCT02178722). Patients previously treated with anti-PD-1 or anti-CTLA-4 therapies were excluded. Enrollment is complete in the epacadostat 25 mg BID, 50 mg BID, and 100 mg BID cohorts with pembrolizumab 2 mg/kg IV q3 weeks. Expansion cohorts of epacadostat 50 mg BID, 100 mg BID, and 300 mg BID with pembrolizumab 200 mg are enrolling. Safety, tolerability, and investigator-assessed tumor response (RECIST 1.1) were evaluated.

## Results

As of August 21, 2015, 54 patients were enrolled. This report includes safety data on 28 patients (melanoma [n=11], RCC [n=5], NSCLC [n=5], TCC [n=3], EA and SCCHN [n=2 each]) and 19 patients evaluable for efficacy as of July 13, 2015. A DLT (grade 3 rash) was observed in 1/8 patients with epacadostat 50 mg BID/pembrolizumab 2 mg/kg; no DLTs were observed with epacadostat 100 mg/pembrolizumab 2 mg/kg. The most common (≥20%) all grade AEs were fatigue, diarrhea, rash, arthralgia, and nausea; the majority of these were grade 1 or 2. Grade ≥3 immune-related AEs were mucosal inflammation and rash (n=1 [4%] each). Reductions in tumor burden were observed in 15/19 evaluable patients. Responses were observed in all tumor types (Table 1), and all are ongoing. In 7 evaluable melanoma patients, ORR was 57% and DCR was 86%, which included 2 CRs. In 5 evaluable RCC patients, ORR was 40% and DCR was 80%. Based on a PK-PD model for epacadostat, nearly all patients' C_avg_ exceeded the IC_50_, the range of active drug exposure seen in preclinical models.

**Table 1:** 

Evaluable patients*	Melanoma	RCC	TCC	NSCLC	EA	SCCHN
n(%)	(n=7)	(n=5)	(n=2)	(n=2)	(n=2)	(n=1)
**ORR (CR+PR)**	**4 (57)**	**2 (40)**	**1 (50)**	**1 (50)**	**1 (50)**	**1 (100)**
CR	2 (29)	0	0	0	0	0
PR	2 (29)	2 (40)	1 (50)	1 (50)	1 (50)	1 (100)
SD	2 (29)	2 (40)	0	1 (50)	0	0
**DCR (CR+PR+SD)**	**6 (86)**	**4 (80)**	**1 (50)**	**2 (100)**	**1 (50)**	**1 (100)**
PD	1 (14)	0	1 (50)	0	0	0
Not assessable	0	1 (20)	0	0	1 (50)	0

*Patients with ≥ 1 post-baseline response assessment or discontinued from study or died before response could be assessed.

## Conclusions

Epacadostat with pembrolizumab was generally well tolerated and efficacy data suggest promising clinical activity. Correlations between biomarker expression and response are being evaluated. Enrollment in expansion cohorts is ongoing. Updated data will be presented.

## References

[B1] Gibney GT European Cancer Congress 2015 [abstract 511]

